# Klotho expression is a prerequisite for proper muscle stem cell function and regeneration of skeletal muscle

**DOI:** 10.1186/s13395-018-0166-x

**Published:** 2018-07-04

**Authors:** Hellen E. Ahrens, Judith Huettemeister, Manuel Schmidt, Christoph Kaether, Julia von Maltzahn

**Affiliations:** 1 Leibniz-Institute on Aging – Fritz-Lipmann-Institute, Beutenbergstrasse 11, 07745 Jena, Germany; 2 Present address: Max-Delbrück-Center for Molecular Medicine, Robert-Rössle-Str. 10, 13125 Berlin, Germany

**Keywords:** Skeletal muscle, Regeneration, Klotho, Myogenesis, Muscle stem cell, Aging, Canonical Wnt signaling, Wnt3a

## Abstract

**Background:**

Klotho is a well-known anti-aging hormone, which serves as a suppressor of aging through a variety of mechanisms. Aging of skeletal muscle is concomitant with a decrease in muscle stem cell function resulting in impaired regeneration.

**Methods:**

Here we investigate the functional role of the anti-aging hormone Klotho for muscle stem cell function after cardiotoxin-induced injury of skeletal muscle using a klotho hypomorphic mouse line, which is characterized by a premature aging phenotype. Furthermore, we perform floating single myofiber cultures with their adjacent muscle stem cells to investigate the interplay between canonical Wnt signaling and Klotho function.

**Results:**

We demonstrate that muscle stem cell numbers are significantly decreased in klotho hypomorphic mice. Furthermore, we show that muscle stem cell function is also severely impaired upon loss of klotho expression, in culture and during regeneration in vivo. Moreover, we demonstrate that addition of recombinant Klotho protein inhibits aberrant excessive Wnt signaling in aged muscle stem cells thereby restoring their functionality.

**Conclusions:**

The anti-aging hormone Klotho counteracts aberrant canonical Wnt signaling in muscle stem cells and might be one of the naturally occurring inhibitors of canonical Wnt signaling in skeletal muscle.

**Electronic supplementary material:**

The online version of this article (10.1186/s13395-018-0166-x) contains supplementary material, which is available to authorized users.

## Background

Skeletal muscle is one of the tissues with the highest ability to regenerate [1]. Muscle stem cells, also named satellite cells, are a prerequisite for functional regeneration [2, 3]. Under resting conditions they are quiescent and located under the basal lamina [4]. After injury, muscle stem cells become activated, proliferate, and differentiate to form new myotubes [5]. Muscle stem cell functionality can be affected by intrinsic changes in the cells themselves and changes in the environment—extrinsic changes [1, 4]. Diseases such as muscular dystrophy and other conditions such as natural aging are major factors influencing muscle stem cell function [6–8]. However, during aging, the regenerative capacity of skeletal muscle decreases dramatically [7, 9–12]. Furthermore, muscle stem cell number and functionality decline with age caused by intrinsic and extrinsic changes [8]. Interestingly, several studies demonstrated constant upregulation of a number of developmental signaling pathways such as JAK/STAT and Hox signaling in aged muscle stem cells [7, 11, 12]. Additionally it is known that systemic factors and changes in the muscle stem cell niche can influence regeneration of skeletal muscle in the aged [10].

*Klotho* is a well-known anti-aging gene coding for the αklotho protein, which serves as a suppressor of aging through a variety of mechanisms [13]. Two transcripts can arise from the *klotho* gene through alternative splicing. Thereby, a membrane protein (mKlotho) and a secreted protein are generated, of which the latter one is lacking the transmembrane domain [14]. Furthermore, mKlotho can be cleaved and shedded by secretases, and this form of klotho is referred to as the soluble klotho. The secreted protein and the soluble klotho are here both referred to as sKlotho. Importantly, the different forms of klotho (soluble and membrane bound) inhibit or activate different signaling pathways [13, 14]. mKlotho serves as an obligate co-receptor for fibroblast growth factor 23 (FGF23) in many tissues, e.g., the kidney [15]. There, FGF23 signaling inactivates 1,25-dihydroxyvitamin D3 synthesis and inhibits phosphate reabsorption via ion channel NaPi2a, thus regulating mineral homeostasis [16]. The soluble form of Klotho is mostly shed into the circulation where it interacts with different signaling pathways in the target organs. For instance, sKlotho is known to inhibit insulin/IGF-1 signaling [17]. Furthermore it was demonstrated that sKlotho can inhibit Wnt/β-catenin and TGF-β signaling and might serve as a potential tumor suppressor [18].

Interestingly, serum levels of soluble Klotho decline with age in mice and men [19, 20]. This is in line with reports on klotho hypomorphic mice (ΔKlotho), a well-established model of premature aging [21]. Those mice are genetically characterized by an insertional mutation in the promoter of the *klotho* gene leading to a severe hypomorphic variant through reduced transcription of the *klotho* gene [21]. Mice, which are homozygous for this mutation develop multiple signs of aging including reduced life span, kyphosis, osteoporosis, and arteriosclerosis. Klotho hypomorphic mice are indistinguishable from their wild type littermates until weaning (p21, postnatal day 21) but then rapidly develop a premature aging phenotype with reduced growth, kyphosis, and osteoporosis. Around postnatal day 40 (p40), the aging phenotype is fully developed [21]. Conversely, klotho overexpression leads to an increased lifespan in mice by up to ~ 20–30% [22]. Klotho is predominantly expressed in the kidney, the parathyroid gland, and the cerebral choroid plexus, but also in other organs including skeletal muscle [23].

So far, little is known about the expression and function of klotho in the skeletal muscle. mRNA transcript was detected in lysates from the whole skeletal muscle [21] while the cell type/cell types expressing klotho and its function are still unknown. A study by Phelps et al. in 2013 demonstrated that muscle strength and running endurance are significantly decreased in klotho hypomorphic mice when compared to wildtype littermates [24]. So far, the underlying cause of this decline in muscle strength still needs to be identified. The process of muscle regeneration is fine-tuned and depending on muscle stem cells, which are affected by intrinsic factors in muscle stem cells themselves as well as by systemic effects and factors coming from the stem cell niche [1]. One of the signaling pathways affecting regeneration of skeletal muscle is canonical Wnt signaling described to be increased in aged skeletal muscle [25]. sKlotho is a known inhibitor of canonical Wnt signaling. Therefore, we investigated the effect of Klotho on regeneration of the skeletal muscle, muscle stem cell function, and the interplay between canonical Wnt signaling and sKlotho in muscle stem cells.

We show that klotho hypomorphic mice display disturbed muscle stem cell function as well as reduced regenerative capacity. Furthermore, we identify sKlotho as one of the modulators of muscle stem cell function and thereby regeneration of skeletal muscle, potentially by inhibiting aberrant canonical Wnt signaling, e.g., in the context of aging.

## Methods

### Mice

Klotho deficient (ΔKlotho) mice used in this study were the original hybrid klotho mutant mice backcrossed to 129Sv inbred mice for more than nine generations as described previously [21]. Wildtype and heterozygous littermates served as controls. The C57BL/6J mice used for myofiber culture experiments were obtained from Janvier. Mice were kept in an SPF facility with food and water ad libitum and a fixed 12-h day/night light cycle. All animal experiments were performed in accordance with the German Animal Welfare Act and approved by the responsible local authority of Thuringia (TLV), TVA no.: 03-11/14.

### Muscle injury

Mice were anesthetised with isoflurane. The right hind limb was shaved and disinfected before 50 μl cardiotoxin (10 μM in 0.9% NaCl, Sigma) were injected into the tibialis anterior muscle using a 29 gauge needle as described previously [26]. Analgesics (meloxicam 1 mg/kg body weight) were applied for 3 days. Animals were sacrificed 10 or 21 days after muscle injury.

### Immunofluorescence and immunoblot analyses

Tibialis anterior (TA) and extensor digitorum longum (EDL) muscles were isolated, embedded in OCT (Tissue Tec) containing 10% sucrose and snap-frozen in liquid nitrogen.

Immunofluorescence on thin cryosections (12 μm) was performed after fixation with 2% PFA, permeabilisation (0.1% TritonX100, 0.1 M glycine in phosphate buffered saline (PBS)) and blocking for 1 h at RT in 2.5% mouse-on-mouse (M.O.M.) blocking solution (Vector labs) in PBS. Primary myoblasts, differentiated myotubes, and myofibers were fixed with 2% PFA, permeabilised, and blocked with 5% horse serum in PBS for 1 h at RT. The following primary antibodies were used: anti-Ki67 (rabbit, 1:1000, ab15580, AbCam), anti-Laminin (rabbit, 1:1000, Sigma), anti-MyoD (rabbit, 1:250, Santa Cruz; rat, 1:200 Merck), anti-Pax7 (mouse IgG1, undiluted, Developmental Studies Hybridoma bank (DSHB)), anti-myogenin (mouse IgG1, F5D, undiluted, DSHB), anti-myosin heavy chain (MHC) (mouse IgG2b, MF20, undiluted, DSHB), anti-MHC type IIa (mouse IgG1, SC-71, undiluted, DSHB), anti-MHC type IIx (mouse IgM, 6HI, undiluted, DSHB), anti-MHC type IIb (mouse IgM, BF-F3, undiluted, DSHB), anti-developmental MHC (devMHC) (mouse IgG1, 1:500, BF-45, DSHB), and anti-CD68 (rat, 1:500, Biorad MCA1957). No fixation with PFA was used for devMHC stainings.

The following secondary antibodies were used: anti-rabbit IgG (Alexa-Fluor 488), anti-rabbit IgG (Alexa-Fluor 647), anti-mouse IgG1 (Alexa-Fluor 546 and Alexa 488), anti-mouse IgG2b (Alexa-Fluor 488), and anti-mouse IgM (Alexa-Fluor 488 and Alexa-Fluor 546) (all obtained from Life Technologies, all diluted 1:1000). Nuclei were counterstained with DAPI (1:5000) before mounting with Permafluor (Thermo Scientific).

Immunoblot analyses were performed using the following antibodies: MuRF1/2/3 (rabbit, 1:1000, ab172479, AbCam), Atrogin/Fbxo32 (rabbit, 1:1000, ab168372, AbCam), non-phospho (active) β-Catenin (Ser33/37/Thr41) (D13A1) (rabbit, 1:1000, 8814S, Cell Signaling), phospho (inactive) β-Catenin (Ser33/37/Thr41) (rabbit, 1:1000, 9561S, Cell Signaling), Klotho (goat, 1:2000, AF1819, R&D Systems), Tubulin (mouse, 1:500, T9026, Sigma-Aldrich), GAPDH (G-9) (mouse, 1:200, sc-365062, Santa Cruz), peroxidase goat anti-rabbit (1:1000, P0448, Dako), peroxidase rabbit anti-goat (1:1000, P0449, Dako), and peroxidase goat anti-mouse (1:1500, P0447, Dako), including Pierce™ ECL Western blotting substrate (32106, Thermo Scientific) and Immobilon™ Western chemiluminescent HRP substrate (WBKLS0500, Millipore). Images were acquired with a MyECL Imager (Thermo Scientific).

### Histochemistry

Hematoxylin-Eosin (H&E) stainings of TA cross-sections were performed automatically with the Leica stainer XL as described earlier [26]. Sirius Red, Oil RedO, and Alizarin Red stainings were performed as described earlier [26].

### Muscle stem cell isolation

Complete hind limb musculature was minced and incubated with collagenase B (2.5 g/ml, Roche) and dispase II (1 g/ml, Roche) for 30 min at 37 °C. The digested muscles were further homogenized by trituration and filtered through 74-μm cell filters. Isolated cells were pelleted at 450×*g* and gently resuspended in 500 μl Magnetic-activated cell sorting (MACS) buffer (0.5% BSA, 2 mM EDTA in PBS) followed by addition of 60-μl microbeads coupled to monoclonal antibodies against non-target cells according to instructions provided by the manufacturer (Satellite Cell Isolation Kit, Miltenyi Biotec GmbH). After incubation for 30 min on ice, cells were filtered through a 50-μm filter and loaded on a MACS column (Miltenyi Biotec). The flow-through was collected, washed, and plated on collagen-coated 10-cm cell culture dishes. Staining for Pax7 was performed to check for purity of the isolated cells.

### Cell culture

Primary myoblasts were kept in Ham’s F-10 Nutrient Mix supplemented with 20% fetal bovine serum, 2% penicillin/streptomycin, and 2.5 ng/ml bFGF (Gibco) at 37 °C and 5% CO2. For the proliferation assay, cells were washed in PBS, trypsinized, and seeded onto collagen-coated 24-well plates (25,000 cells per well). After 48 h, cells were fixed with 2% PFA for 5 min followed by subsequent immunofluorescence staining. For differentiation assays cells (33,000 cells per 24-well) were cultured for 3 or 5 days in DMEM containing 2% horse serum and 2% penicillin/streptomycin. Concentration of cell culture supernatants was performed using Amicon columns with a cut-off of 30 kDA as described earlier [27].

### EDL single fiber culture

Single myofibers were isolated from EDL muscle by digestion in DMEM with 0.2% collagenase (from *Clostridium hystolyticum*, Sigma) and cultured in DMEM supplemented with 20% FBS and 1% chick embryo extract (US biological) as described previously [27].

Myofibers of old (22–24 months) and young (2–4 months) C57BL6/J mice were incubated with recombinant human soluble Klotho protein (100 ng/ml, tebu-bio, Cat# enz-369), recombinant mouse Wnt3a (100 ng/ml, R&D Systems, # P27467), recombinant mouse Dkk1 (100 ng/ml, R&D Systems, # O54908), or different combinations of those.

### RNA isolation, cDNA synthesis, and quantitative real-time PCR (qRT-PCR)

Total RNA was isolated from freshly isolated or cultured (72 h) EDL-derived myofibers and kidney samples by using the peqGOLD TriFast (VWR) according to the manufacturer’s instructions. cDNA was synthesized with the iScript cDNA Synthesis Kit (BioRad) according to manufacturer’s instructions. Quantitative real-time PCR was performed in technical triplicates using the iQ SYBR Green Supermix (BioRad) on the Mx3000P qPCR System (Agilent Technologies). The following program was used: 3 min at 95 °C; 40 cycles of 95 °C for 15 s, followed by 58 °C for 15 s, and 72 °C for 30 s; and a final cycle of 95 °C for 15 s, followed by 58 °C for 15 s, and 95 °C for 30 s. qPCR primers for mouse alpha-Klotho detection were purchased from Genecopoeia (Product ID MQP031135). For normalization, we used GADPH primers with the following sequence (5′–3′): GAPDH_USP (forward): ATGCCAGTGAGCTTCCC and GAPDH_DSP (reverse) CATCACCATCTTCCAGGAGC. We calculated relative mRNA expression values with the ΔΔCt method: ∆Ct = Ct (αKlotho) − Ct (GAPDH), relative expression = 2^(−ΔCt)^.

### Digital image acquisition and processing

Images were acquired with the Axio Imager and Axio Observer.Z1 (Carl Zeiss). Immunofluorescence images from whole muscle cross-sections were generated by using the tile mode at 20-fold magnification with the ZEN software (Carl Zeiss). Representative image details were selected from whole cross-section tiled images. The minimal fiber feret of myofibers was determined as the closest distance between two parallel tangents of the fiber [28].

### Statistical analysis

Data were analyzed with the GraphPad Prism software. Statistical significance was determined by an unpaired, one-tailed Student’s *t* test. Results are presented as mean ± standard error of the mean. The calculated *P* values are shown as *, **, and *** which represent *P* < 0.05, *P* < 0.01, and *P* < 0.001, respectively.

## Results

### Muscle stem cell numbers are reduced in ΔKlotho mice

**Fig. 1:**
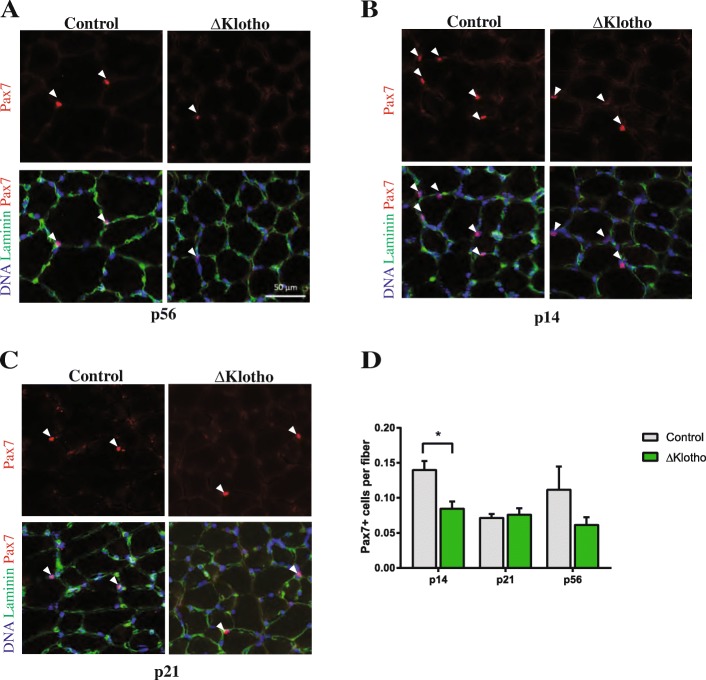
Muscle stem cell numbers are reduced in ΔKlotho mice. **a**–**c** Representative immunofluorescence images of TA muscle cross-sections from ΔKlotho and control mice stained for DAPI (DNA, blue) and Laminin (green), and Pax7 (red) at **a** p56, **b** p14, and **c** p21. Scale bar = 50 μm. **d** Quantification of Pax7-positive SCs on whole TA cross-sections from ΔKlotho and control mice at p14, p21, and p56. *n* = 3 mice per genotype and time point. All data are presented as means ± SEM. *p < 0.05

Several reports suggested that stem cell function in different organs is perturbed in ΔKlotho mice, e.g., adipogenic stem cells [29]. We first asked whether the muscle stem function is also affected in ΔKlotho mice. Therefore, we investigated if the number of muscle stem cells shows differences when comparing muscles from ΔKlotho mice and their littermate controls. Indeed, we found a reduction in the number of muscle stem cells in ΔKlotho mice at p56 and p14 while numbers were comparable at p21, a time point when skeletal muscle matures (Fig. 1a–d). Furthermore, we found an age-dependent difference in myofiber size between ΔKlotho and control mice (Additional file 1: Figure S1A–C). Of note, at p14, the size of myofibers of ΔKlotho and control littermates did not differ. With age, the average fiber feret in muscles of control mice increased at p21 and p56 as expected. In contrast, the average fiber feret from ΔKlotho mice did not increase with age (Additional file 1: Figure S1B). Accordingly, the size of tibialis anterior muscle cross sectional area demonstrated differences at p56 between ΔKlotho and control littermates (Additional file 1: Figure S1C). These age-dependent changes in myofiber size could be either due to a defect in muscle growth or due to a premature aging phenotype which would be concomitant with increased expression of atrophy markers, e.g., MuRF1-3 and Fbxo32/Atrogin/MAFbx [30–33]. Therefore, we investigated the expression of the ubiquitin ligases MuRF1-3 and Fbxo32/Atrogin/MAFbx in ΔKlotho and age-matched control littermates (Additional file 1: Figure S1D). Here, we found that expression of all ubiquitin ligases was increased in ΔKlotho muscles compared to age-matched littermate controls (Additional file 1: Figure S1E). Furthermore, TA muscles are lighter in ΔKlotho mice (Additional file 1: Figure S1F) also when the muscle weight is normalized to the tibia length (Additional file 1: Figure S1G). This is suggesting that muscles from ΔKlotho mice are more likely undergoing a sarcopenia-like muscle wasting than a developmental defect, while also a combination of both is possible. When analyzing the fiber type distribution in EDL muscles between ΔKlotho and control littermates with an age of p56, we did not observe any differences (Additional file 2: Figure S2A–C). The reduced myofiber size in ΔKlotho mice at an age of p56 concomitant with the increased expression of the ubiquitin ligases (MuRF1-3 and Fbxo32/Atrogin/MAFbx) in muscles from ΔKlotho mice suggest that ΔKlotho mice display an age-dependent reduction in muscle mass, which is reminiscent of sarcopenia. This decreased muscle mass is accompanied by reduced numbers of muscle stem cells, which might lead to impaired regeneration of skeletal muscle.

### Regeneration of skeletal muscle is severely impaired in ΔKlotho mice

**Fig. 2:**
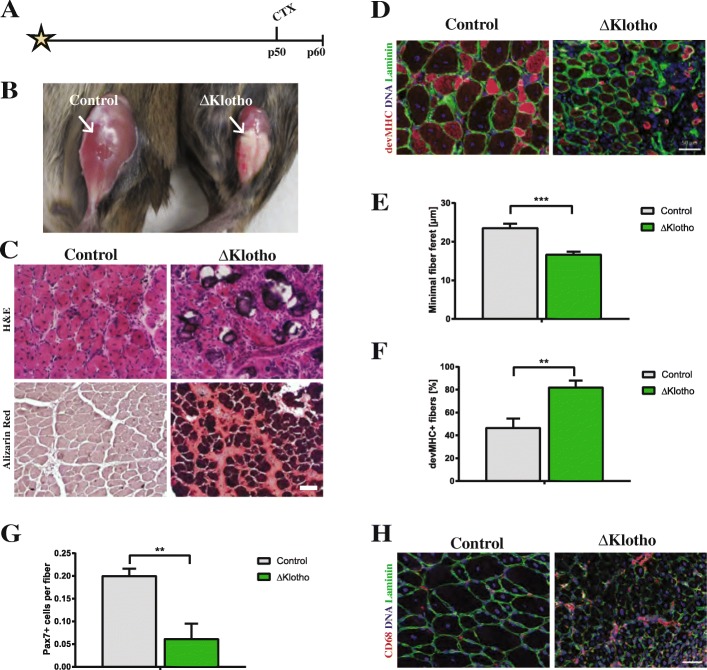
Early regeneration of skeletal muscle is impaired in ΔKlotho mice. **a** The right TA muscle from ΔKlotho and control mice was injured by injection of cardiotoxin (CTX) at p50 and isolated at p60. **b** TA muscles (arrows) show extensive fibrosis in ΔKlotho mice 10 days (d10) after injury. **c** Representative H&E stainings and Alizarin Red stainings of TA muscle cross-sections at d10 after injury. Necrotic tissue, fatty vacuoles, and massive cell invasion are visible in ΔKlotho TA muscles, while control TA muscles show mostly regenerating myofibers. Scale bar = 100 μm. **d** Immunostainings of cross-sections of TA muscles at d10 after injury with antibodies directed against Laminin (green) and developmental (dev) MHC (red), nuclei are stained with DAPI (DNA, blue). Scale bar = 50 μm. **e** Minimal fiber feret measured on whole cross-sections from ΔKlotho and control TA muscles at d10 after injury. (ΔKlotho *n* = 4 mice, control *n* = 5 mice). Proportion of **f** devMHC-positive fibers and **g** Pax7-positive cells per myofiber on whole cross-sections at d10 after CTX-induced injury (ΔKlotho *n* = 3 mice, control *n* = 4 mice). **h** Representative images of stainings for CD68 (in red) and laminin (in green) showing increased infiltration with macrophages in regenerating muscles from ΔKlotho mice. Scale bar = 50 um. All data are presented as means ± SEM. *p < 0.05, **p < 0.01, ***p < 0.001

Impaired muscle stem cell function generally coincides with decreased regenerative capacity of skeletal muscle [4, 34]. To analyze the role of klotho during regeneration, we injured the tibialis anterior muscles of ΔKlotho and control mice at an age of p50 and analyzed the regeneration 10 days after injury (Fig. 2a). Intriguingly, we already observed obvious impairments in the regeneration process in muscles from ΔKlotho mice on the macroscopic level (Fig. 2b). Muscles from control littermates after 10 days of regeneration showed the expected degree of regeneration (d10) (compare [34]). In contrast, ΔKlotho mice exhibited signs of fibrosis, calcification, and scarring suggesting major impairments in regeneration, confirmed by histological analyses (Fig. 2c). To quantify regeneration, we measured the minimal fiber feret as a measure of myofiber size. Thereby, we found significantly smaller myofibers after d10 of regeneration in ΔKlotho mice (control 23.58 ± 1.08 μm vs. ΔKlotho 16.70 ± 0.71 μm) (Fig. 2d, e). This suggests either impaired differentiation of muscle stem cells or loss of self-renewal capacity thereby limiting the number of stem cells available for formation and growth of newly formed fibers. To further investigate the differentiation process in vivo, we quantified the number of devMHC (developmental myosin heavy chain) positive myofibers (Fig. 2d, f), a well-known marker for newly formed myofibers. Importantly, during maturation of myofibers, expression of devMHC decreases [35]. We found that 82% of myofibers in ΔKlotho mice still expressed devMHC while only 47% of myofibers in the control littermates were positive for devMHC at day 10 after CTX injury. This indicates that differentiation/maturation of myofibers is delayed/inhibited in ΔKlotho muscles (Fig. 2d, f). Muscle stem cells can divide asymmetrically during the regeneration process thereby generating progenitor cells to build new myofibers or self-renew to replenish the pool of stem cells [25]. To investigate the self-renewal potential of muscle stem cells, we counted the number of muscle stem cells (Pax7 positive) at day 10 after injury. Here, we found a dramatic reduction of 69% in muscle stem cell numbers in ΔKlotho muscles (control 0.200 ± 0.016 Pax7+ cells/fiber vs. ΔKlotho 0.062 ± 0.033 Pax7+ cells/fiber; Fig. 2g) while numbers of macrophages were increased in ΔKlotho mice (Fig. 2h). These results suggest that differentiation/maturation of newly formed myofibers and self-renewal of muscle stem cells are severely compromised.

**Fig. 3:**
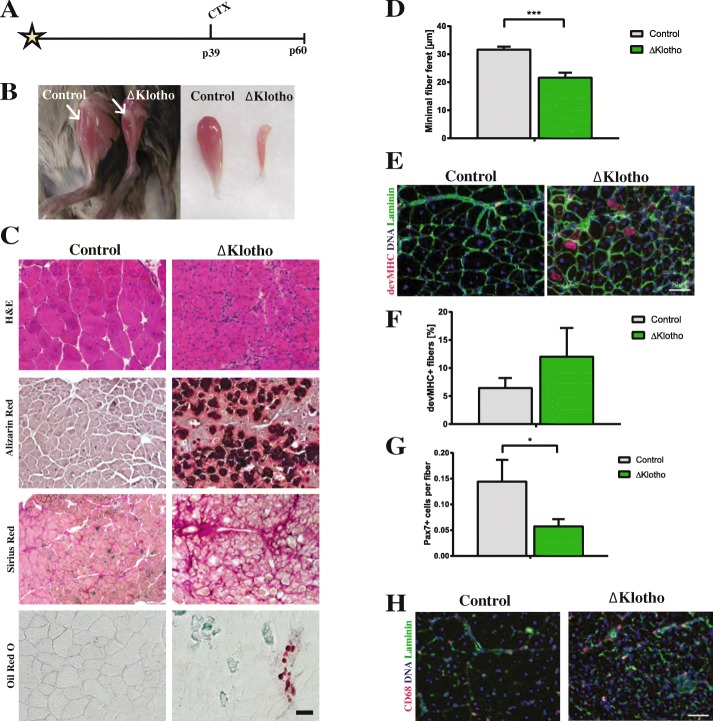
Late regeneration of skeletal muscle is impaired in ΔKlotho mice. **a** The right TA muscle from ΔKlotho and control mice was injured by injection of cardiotoxin (CTX) at p39 and isolated at p60. **b** TA muscles (arrows) show signs of impaired regeneration by H&E staining and extensive fibrosis in ΔKlotho mice at day 21 after injury (**c**), but not in control littermates as demonstrated by Sirius Red staining. Alizarin Red marks calcifications, visible in regenerating muscles from ΔKlotho mice as well as lipid accumulations shown by Oil Red O staining. Scale bar = 100 μm. **d** Minimal fiber feret measured on whole cross-sections of TA muscles from ΔKlotho and control at d21 after injury. (ΔKlotho *n* = 4 mice, control *n* = 4 mice). **e** Immunostainings of cross-sections of TA muscles at d21 after injury with antibodies directed against laminin (green) and developmental (dev) MHC (red), nuclei are stained with DAPI (DNA, blue). Scale bar = 50 μm. Proportion of (**f**) devMHC-positive fibers (ΔKlotho *n* = 2 mice, control *n* = 4 mice) and (**g**) Pax7-positive satellite cells per myofiber on whole cross-sections at d21 after CTX-induced injury. (ΔKlotho *n* = 4 mice, control *n* = 4 mice). **h** Representative images of stainings for CD68 (in red) and laminin (in green) showing increased infiltration with macrophages in regenerating muscles from ΔKlotho mice. Scale bar = 50 um. All data are presented as means ± SEM. *p < 0.05, **p < 0.01, ***p < 0.001

To further address the self-renewal ability of muscle stem cells in vivo, we investigated regeneration of skeletal muscle 21 days after injury (Fig. 3a), a time point when the regeneration process is nearly completed and muscle stem cells start to become quiescent again [4]. Gross morphological impairments in muscle regeneration were also obvious 21 days after injury in ΔKlotho mice (Fig. 3b), again showing scarring, aberrant calcification, accumulation of lipids, and signs of fibrosis as observed 10 days after injury (Fig. 2b, c). Histological analyses demonstrated that the regeneration process in ΔKlotho mice was still strongly impaired compared to littermate controls with noticeably smaller myofibers and increased numbers of mononucleated cells at day 21 after injury (Fig. 3c) suggesting either increased infiltration of immune cells or lack of differentiation of myogenic cells. For quantification of the regeneration process, we measured the minimal fiber feret and found also here a significant decrease (32%) in myofiber size in muscles from ΔKlotho mice compared to littermate controls (control 31.77 ± 0.97 μm vs. ΔKlotho 21.75 ± 1.62 μm; Fig. 3d). To get further insights into the maturation process of newly formed myofibers, we counted the number of devMHC-positive myofibers at day 21 after injury (Fig. 3f). In control muscles, we hardly found any devMHC-positive myofibers as expected and described in the literature [35]. Consistent with our previous observations, the percentage of devMHC myofibers in ΔKlotho muscles was increased by nearly twofold (control 6.49 ± 1.73% vs. ΔKlotho 12.08 ± 5.083%; Fig. 3f) suggesting impaired differentiation/maturation of myofibers in ΔKlotho mice after injury. When we counted the number of Pax7-positive muscle stem cells 21 days after regeneration, we found a dramatic decrease in ΔKlotho mice compared to littermate controls (60% difference; Fig. 3g) indicating severe impairments in self-renewal of muscle stem cells when klotho expression is lost. Furthermore, numbers of macrophages were still increased at day 21 after regeneration in ΔKlotho mice (Fig. 3h).

### Loss of mklotho does not affect myogenic proliferation or differentiation

**Fig. 4:**
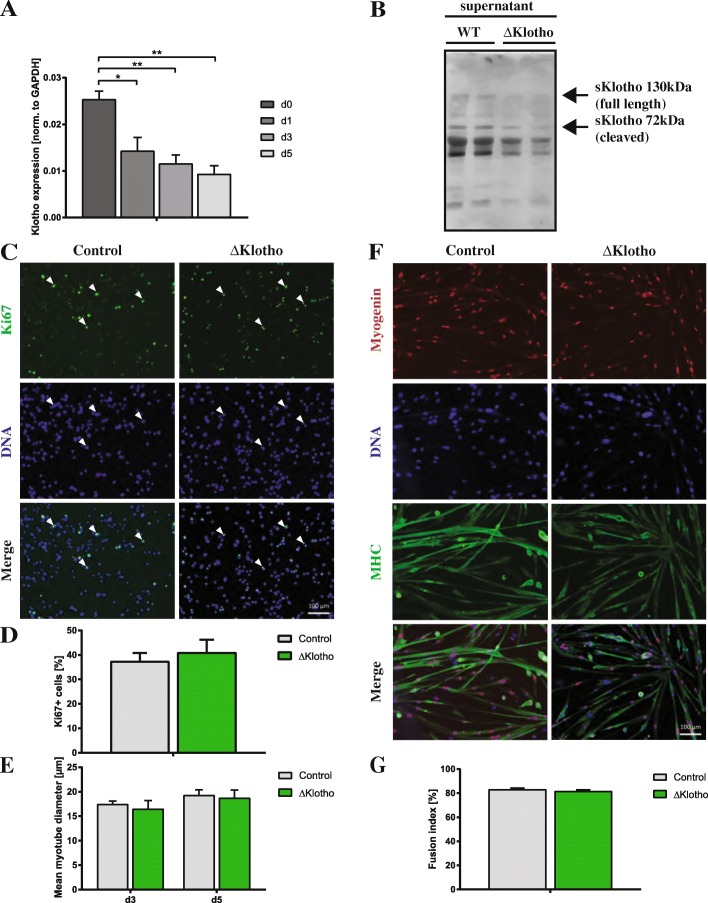
Proliferation and differentiation are not affected in myoblasts from ΔKlotho mice in vitro. **a** *αklotho* mRNA expression during myogenic differentiation of myoblasts d0, growth medium (myoblasts); d1, day1 of differentiation (myocytes); d3 and d5, days 3 and 5 of differentiation (myotubes). (*n* = 3 mice per time point). All data are presented as means ± SEM. *p < 0.05, **p < 0.01. Expression was normalized to GAPDH. **b** Immunoblot analysis showing expression of sKlotho in the supernatant from primary myoblasts isolated from wt mice but not in the supernatant from ΔKlotho mice. An image of the Ponceau stained membrane can be found in Additional file 3: Figure S3F. **c** Primary myoblasts from ΔKlotho and control mice were immunostained for the proliferation marker Ki67 (green) and DAPI (DNA, blue) after 48 h of proliferation time in normal culture medium. Arrow heads mark Ki67 positive cells. Scale bar = 100 μm. **d** The proportion of Ki67-positive cells was counted in 15 randomly chosen regions of interest per condition (ΔKlotho *n* = 3 mice, control *n* = 4 mice). **e** The mean myotube diameter was measured in six randomly chosen regions of interest per condition (ΔKlotho *n* = 3 mice, control *n* = 4 mice). **f** Representative images of immunostainings from myotubes after 5 days of differentiation. DNA (DAPI, blue), MHC (green), myogenin (red). Scale bar = 100 μm. **g** The fusion index was calculated as the ratio of nuclei in myotubes in relation to the total number of nuclei in 6 randomly chosen regions of interest per condition (ΔKlotho *n* = 3 mice, control *n* = 4 mice). All data are presented as means ± SEM

In the in vivo model, we used the expression of both forms of klotho (mKlotho and sKlotho) which are ubiquitously perturbed. Furthermore, it is not possible to investigate the role of mKlotho independently of systemic effects including reduced serum levels of sKlotho, which arise from loss of klotho expression in the whole body. To gain information about expression of klotho mRNA (detecting mRNA for mKlotho and sKlotho) in myogenic cells, we performed expression analyses of primary myoblasts and myotubes (Fig. 4a). We can detect klotho mRNA in myoblasts (d0), where expression decreases following differentiation suggesting that expression of klotho mRNA is most important in undifferentiated cells. Furthermore, we asked whether myoblasts secrete sKlotho (Fig. 4b, Additional file 3: Figure S3E, F). Indeed, we found that myoblasts isolated from wt animals secrete small amounts of sKlotho (Additional file 3: Figure S3E), this is lost in primary myoblasts from ΔKlotho mice—as expected (Fig. 4b). We then isolated primary myoblasts from ΔKlotho and control littermates and analyzed their proliferation and differentiation potential (Fig. 4c–g). Importantly, by using this approach, we exclude systemic factors, e.g., changes in the mineral homeostasis of ΔKlotho mice and sKlotho secreted by the kidney and transported to the muscle via the blood stream. Furthermore, we exclude factors from the muscle stem cell niche, e.g., changes in the extracellular matrix. The only difference between the myogenic cells from ΔKlotho and control mice is the expression of mKlotho and secretion of sKlotho by myoblasts or myotubes themselves. Interestingly, no differences in proliferation (Fig. 4c, d) or differentiation (Fig. 4e–g and Additional file 3: Figure S3 A–D) were observed between myogenic cells isolated from ΔKlotho and control littermates implying that expression of mKlotho and secretion of sKlotho in myoblasts does not affect their proliferation and differentiation. Therefore, we can conclude that endogenous expression of mKlotho is dispensable for proper proliferation and differentiation of myoblasts.

### Muscle stem cell function is severely impaired in adult ΔKlotho mice

**Fig. 5:**
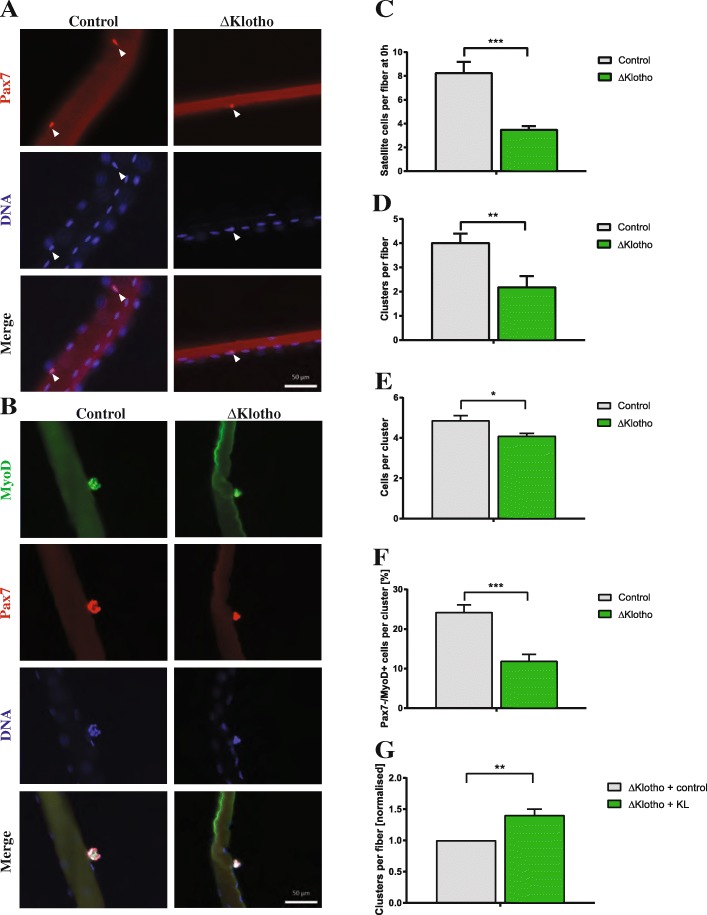
Muscle stem cell function is impaired in ΔKlotho mice. **a** Myofibers with their adjacent muscle stem cells were isolated from EDL muscles of p42 old mice and directly fixed and stained with antibodies directed against Pax7 (red) and DNA (DAPI, blue). Arrows point at Pax7-positive muscle stem cells. Scale bar = 50 μm. **b** Representative images of clusters of muscle stem cells on their adjacent EDL myofibers isolated at p42 and cultured for 72 h and then stained with antibodies directed against MyoD (green), Pax7 (red), and DNA (DAPI, blue). Scale bar = 50 μm. **c** The number of Pax7-positive muscle stem cells per myofiber was quantified from p42 ΔKlotho and control mice. (ΔKlotho *n* = 5 mice, control *n* = 7 mice). **d** The number of clusters per myofiber isolated from p42 animals and cultured for 72 h of culture was enumerated. (ΔKlotho *n* = 5 mice, control *n* = 7 mice). **e** The number of cells per cluster per myofiber isolated from p42 animals and cultured for 72 h was determined. (ΔKlotho *n* = 5 mice, control *n* = 7 mice). **f** The composition of clusters shows a reduction in the proportion of further differentiated cells (Pax7−/MyoD+) in ΔKlotho compared to control mice at p42. (ΔKlotho *n* = 5 mice, control *n* = 7 mice). **g** Addition of recombinant sKlotho to fiber cultures from ΔKlotho mice results in an increase in cluster number per myofiber (*n* = 3). All data are presented as means ± SEM. *p < 0.05, **p < 0.01, ***p < 0.001

To investigate the muscle stem cell function in their endogenous niche but without the influence of systemic factors, we performed single myofiber cultures from EDL muscles [36]. In this culture system, the muscle stem cells are located in their endogenous niche adjacent to the muscle fiber and can undergo multiple rounds of divisions under controlled culture conditions. Therefore, we first compared the number, activation, and differentiation potential of muscle stem cells of ΔKlotho and control mice. We observed a dramatic reduction (58%) in the average number of muscle stem cells per myofiber in ΔKlotho mice directly after isolation (Fig. 5a, c). Importantly, the reduction of muscle stem cells observed in the EDL was similar to the one observed in the TA muscle (Fig. 1a, d). To investigate the proliferative capacity, we determined the number of clusters formed from a single muscle stem cell. Notably, we also observed a dramatic reduction in the average number of clusters per myofiber in ΔKlotho mice compared to littermate controls (Fig. 5b, d) as well as in the average number of cells per cluster (Fig. 5e). This indicates that proliferation and/or differentiation of muscle stem cells are affected in ΔKlotho mice. However, we did not observe differences in the activation potential (Additional file 4: Figure S4A). After muscle injury or culture, muscle stem cells are activated resulting in the expression of MyoD and acquisition of a progenitor status. Subsequent downregulation of Pax7 (Pax7−/MyoD+) is followed by differentiation [37, 38]. Indeed, we found a reduction in the percentage of cells, which only express MyoD in clusters from ΔKlotho mice (Fig. 5f; Additional file 4: Figure S4B–D). This is suggestive of impaired differentiation of muscle stem cells in ΔKlotho mice. Interestingly, the same loss of differentiation can be seen in muscle stem cells cultured on floating myofibers isolated from aged mice [7]. To investigate if recombinant sKlotho (KL) is able to rescue the decrease in cluster formation observed in ΔKlotho mice, we applied sKlotho recombinant protein to fiber cultures from ΔKlotho mice (Fig. 5g). Indeed, addition of sKlotho results in an increase in the number of clusters per fiber of ΔKlotho mice suggesting that sKlotho and not mKlotho is important for muscle stem cell function.

### Wnt3a antagonizes Klotho function in muscle stem cells

Canonical Wnt is one of the secreted factors, which play an important role in stem cell maintenance and stem cell proliferation [39]. In muscle stem cells, canonical Wnt signaling controls differentiation of muscle stem cells; for instance, it was demonstrated that a switch from Notch signaling to canonical Wnt signaling is a prerequisite for the differentiation of muscle stem cells [25, 40, 41]. Although necessary for differentiation, exogenous induction of canonical Wnt signaling during early regeneration of skeletal muscle leads to premature differentiation of progenitor cells thereby depleting the muscle stem cell pool [40]. Furthermore, it was described that addition of canonical Wnt3a to adult skeletal muscle leads to increased amounts of connective tissue resulting in a skeletal muscle, which resembles aged skeletal muscle after regeneration. sKlotho is a known inhibitor of canonical Wnt signaling [42]. The KL1 domain of sKlotho was recently mapped as the interaction domain of sKlotho with Wnt3a [43]. This evidence prompted us to speculate if sKlotho could be an inhibitor of canonical Wnt signaling in muscle stem cells.

**Fig. 6:**
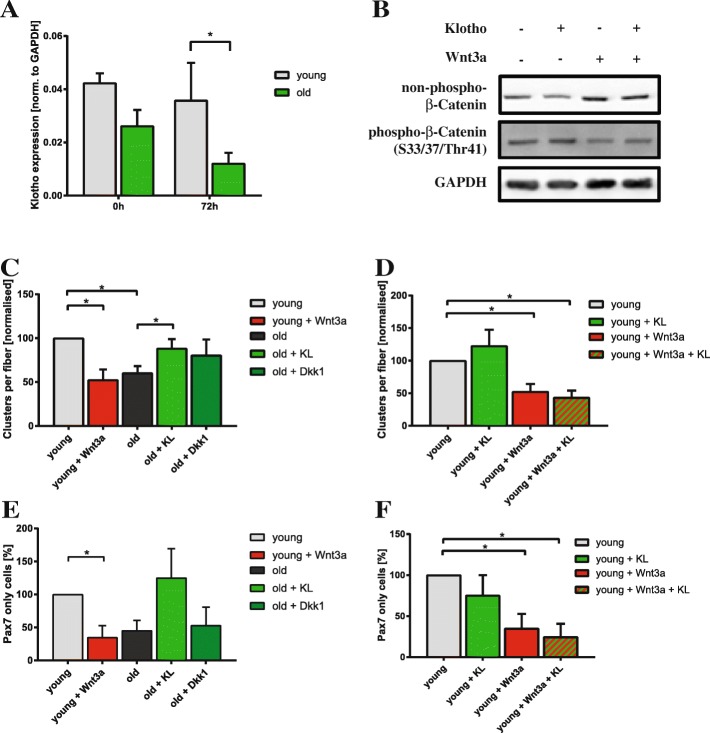
sKlotho antagonizes aberrant Wnt3a function in aged muscle stem cells. **a** *αKlotho* mRNA expression in myofibers with their adjacent muscle stem cells directly after isolation or after 72 h of culture from young and old C57BL/6J mice (young mice: *n* = 4, old mice: *n* = 5). **b** Addition of sKlotho to primary myoblasts reduces induction of canonical Wnt signaling evoked by addition of recombinant Wnt3a. **c** Myofibers with their adjacent muscle stem cells from young (4 months) and old (22–24 months) mice were cultured for 72 h with normal medium, recombinant soluble klotho (KL) protein, recombinant Wnt3a, or recombinant Dkk1. (*n* = 6 mice (young), *n* = 5 (old)). The number of clusters per myofiber was normalized to young control. **d** Myofibers with their adjacent muscle stem cells from young (4 months) mice were cultured for 72 h with normal medium, recombinant soluble klotho (KL) protein, recombinant Wnt3a, or a combination of both (*n* = 6 mice). The number of clusters per myofiber was normalized to young control. **e** Addition of sKlotho recombinant protein (KL) increases the proportion of Pax7+/MyoD− cells per fiber located in a cluster. The number of Pax7+/MyoD− cells (located in a cluster) per myofiber was normalized to young control (*n* = 6 mice (young), *n* = 5 (old)). **f** Myofibers with their adjacent muscle stem cells from young (4 months) mice were cultured for 72 h with normal medium, recombinant soluble klotho (KL) protein, recombinant Wnt3a, or a combination of both (*n* = 6 mice). The number of Pax7+/MyoD− cells (located in a cluster) per myofiber was normalized to young control. All data are presented as means ± SEM. *p < 0.05, **p < 0.01, ***p < 0.001

Therefore, we first evaluated the expression levels of klotho mRNA in isolated myofibers with their adjacent muscle stem cells isolated from young and old C57BL/6J mice (Fig. 6a). Interestingly, expression of klotho mRNA is reduced in myofibers with adjacent muscle stem cells from old mice directly after isolation and after 72 h of culture when the number of muscle stem cells on the myofibers is increased. This let us speculate that less sKlotho is secreted from aged myofibers and their adjacent muscle stem cells thereby impeding muscle stem cell function. To demonstrate that sKlotho can also inhibit canonical Wnt signaling in myogenic cells, we treated primary myoblasts with Wnt3a, sKlotho, or a combination of both (Fig. 6b). Importantly, Wnt3a-mediated activation of canonical Wnt signaling is inhibited by addition of sKlotho suggesting that sKlotho is also an inhibitor of canonical Wnt signaling in myogenic cells (Fig. 6b, Additional file 5: Figure S5A).

### Supplementation with sKlotho rejuvenates aged muscle stem cells

We speculated that sKlotho could be one of the naturally occurring inhibitors of canonical Wnt signaling in muscle stem cells in the young and that loss of klotho expression in the aged might be one of the causes for increased canonical Wnt signaling in aged muscle stem cells. Therefore, we cultured muscle stem cells on floating fibers from young and old mice with different combinations of sKlotho protein (KL), Wnt3a and Dkk1, a well-known inhibitor of canonical Wnt signaling (Fig. 6c). Interestingly, we found that addition of sKlotho protein to fiber cultures obtained from old C57BL/6J mice (22–24 months of age) resulted in an increase in the number of clusters per fiber formed after 72 h of culture and also an increased activation potential (Additional file 5: Figure S5C). Importantly, the number of clusters per fiber was similar to the number of clusters per fiber in young animals (4 months of age) suggesting that sKlotho rejuvenates old muscle stem cells (Fig. 6c). The effect of sKlotho on cluster numbers on aged fiber cultures was similar to the effect of Dkk1. Importantly, addition of sKlotho and Dkk1 to old myofibers did not lead to an increased cluster number compared to old myofibers treated with either sKlotho or Dkk1 alone indicating that sKlotho and Dkk1 are both acting by inhibition of canonical Wnt signaling (Additional file 5: Figure S5B). Addition of Wnt3a to young fibers resulted in a decrease in cluster numbers on young fibers as expected (Fig. 6c). Interestingly, the number of clusters was then similar to the ones on old myofibers. However, if we simultaneously added sKlotho and Wnt3a to the myofiber cultures from young mice, this sKlotho-mediated increase in the number of clusters per fiber was completely blocked suggesting that sKlotho and Wnt3a antagonize each other in muscle stem cells (Fig. 6d).

To address if sKlotho is also affecting self-renewal of muscle stem cells in the aged, we counted the number of Pax7-positive/MyoD-negative self-renewing muscle stem cells in clusters on myofibers of old and young mice treated with Wnt3a or sKlotho (Fig. 6e). sKlotho also increased the number of self-renewing muscle stem cells in the aged to numbers comparable to muscle stem cells on young fibers. Of note, Wnt3a and sKlotho are also counteracting each other regarding the self-renewal of young muscle stem cells (Fig. 6f) and a combined treatment with sKlotho and Dkk1 does not lead to increased self-renewal compared to treatment with either sKlotho or Dkk1 alone (Additional file 5: Figure S5D).

sKlotho might be an effective natural inhibitor of canonical Wnt signaling in muscle stem cells in the young and loss of klotho expression in the aged might be one of the causes for increased canonical Wnt signaling and thereby impeded muscle stem cell functionality in the aged. This, together with the data on supplementation of sKlotho in aged muscle stem cells, suggests that muscle stem cells and myofibers secrete sKlotho thereby inhibiting canonical Wnt signaling, a mechanism which is particularly hampered in the aged.

## Discussion

Klotho hypomorphic mice display several aberrations in muscle function, for instance, reduced muscle strength and running endurance [24]. They also demonstrate reduced serum levels of sKlotho. Interestingly, a study by Semba et al. [44] found that reduced serum levels of sKlotho in older humans is correlated with reduced grip strength further strengthening the importance of constant klotho expression for maintenance and functional output of skeletal muscle. So far, nothing was known regarding effects of klotho expression on muscle stem cell function and differentiation of myoblasts.

Notably, in the current study, we observed that muscle stem cell function in klotho hypomorphic mice is massively perturbed. Furthermore, numbers of muscle stem cells are significantly decreased in ΔKlotho mice (Figs. 1 and 5a, c). Concomitantly, loss of klotho expression leads to a dramatic reduction in the regenerative capacity of skeletal muscle, which reminded us of the phenotype we observed during natural aging (Figs. 2 and 3; [7, 11]). Klotho hypomorphic mice are not only characterized by the loss of klotho gene expression but also by a disturbed mineral homeostasis caused by impaired FGF23 signaling in the kidney [45]. Since we could not rule out the effects of this disturbed mineral homeostasis in ΔKlotho mice, we performed experiments using cultures of muscle stem cells on single floating myofibers. In this system, we can exclude direct effects from the mineral homeostasis but not the fact that those changes might have led to changes in the epigenome or activity of signaling pathways before isolation. Such changes were described for naturally aged muscle stem cells [7, 11]. Of note, Wehling-Henricks and colleagues described that epigenetic silencing of klotho in muscular dystrophy contributes to the disturbed regeneration and fibrosis in mdx mice, the mouse model for Duchenne muscular dystrophy [46].

In our floating fiber culture system, we found that loss of klotho expression reduces the number of clusters formed and the number of cells per cluster suggesting that loss of klotho expression negatively influences proliferation and self-renewal of muscle stem cells (Fig. 5). Furthermore, differentiation of muscle stem cells on fibers is affected when expression of klotho is lost (Fig. 5 and Additional file 4: Figure S4) implying also a function of klotho in differentiation of muscle stem cells. In adipose-derived stem cells, loss of klotho expression hinders proliferation and differentiation [29]. Application of recombinant sKlotho protein on the other hand restored the proliferative capacity as well as the ability to differentiate. In the skin and intestine, loss of klotho results in exhaustion of the stem cell pool [42].

Canonical Wnt signaling plays an important role in muscle stem cell function [25]. Differentiation of muscle stem cells is controlled by canonical Wnt signaling, especially by signaling through the ligand Wnt3a. Although necessary for the differentiation process, aberrant canonical Wnt signaling results in increased fibrosis of skeletal muscle, for instance, in the context of aging or muscular dystrophy [40, 47]. Aged skeletal muscle has been shown to display increased canonical Wnt signaling resulting in a conversion of muscle stem cells from a myogenic to a fibrogenic lineage [40]. sKlotho is a known antagonist of canonical Wnt signaling [42]. Therefore we investigated if addition of sKlotho protein counteracts aberrant canonical Wnt signaling, here in the context of aging. Indeed, we found that Wnt3a antagonizes sKlotho function in myoblasts and old muscle stem cells (Fig. 6c–f). Furthermore we could demonstrate that inhibition of aberrant canonical Wnt signaling in the aged by Dkk1 had very similar effects compared to addition of sKlotho further supporting the notion that sKlotho is a naturally occurring inhibitor of canonical Wnt signaling in muscle stem cells.

We consider it likely that the large quantity of fibrotic tissue during impaired regeneration and the depletion of muscle stem cells in ΔKlotho mice can be traced back to chronic canonical Wnt stimulation as observed during natural aging [40]. Transforming growth factor beta (TFG-β1) signaling is another pro-fibrotic pathway increased in skeletal muscle during aging resulting in reduced regenerative capacity [48]. Interestingly, sKlotho is a direct inhibitor of TGF-β1 signaling by binding to the type-II TGF-β receptor thereby preventing fibrosis [49]. Therefore, it is likely that sKlotho not only inhibits canonical Wnt signaling but also TFG-β1 signaling in aged skeletal muscle thereby maintaining muscle stem cell function.

Importantly, restoration of sKlotho levels in the aged muscle stem cell niche improves muscle stem cell function in the aged. This suggests that acute sKlotho availability is dominant over intrinsic changes in aged muscle stem cells, which have been acquired over life time [7, 11, 12].

## Conclusions

We conclude that reduction of sKlotho is an important contributor to loss of muscle stem cell function in the aged, most likely through loss of inhibition of canonical Wnt signaling. Therefore, replenishing the levels of sKlotho is a promising therapeutic approach for stimulation of muscle regeneration in the aged.

## Additional files

Additional file 1: **Figure S1.** ΔKlotho mice display signs of sarcopenia. (A) Immunofluorescence of cross-sections of TA muscles from ΔKlotho and control mice stained for DAPI (DNA, blue) and Laminin (green) at p14, p21, and p56. Scale bar = 50 μm. (B) Minimal fiber feret measured on the whole cross-sections from ΔKlotho and control TA muscles at p14, p21, and p56. (p14, p56 *n* ≥ 3 mice per genotype, p21 *n* = 3 mice per genotype). (C) Quantification of the cross-section area of the mid-belly region of TA muscles determined from sections from ΔKlotho and control at p14, p21, and p56. (p14, p56 *n* ≥ 3 mice per genotype, p21 *n* = 3 mice per genotype). (D) Immunoblot analyses of atrophy associated ubiquitin ligases in TA muscles from ΔKlotho and control littermates at different ages. (E) Fold expression of different ubiquitin ligases as shown in (D), values for ΔKlotho are normalized to control animal of the same age. (F) Muscle weight of tibialis anterior (TA) muscles from adult ΔKlotho (*n* = 6) and control mice, *n* = 8 (G) Muscle weight of tibialis anterior (TA) muscles normalized to the length of the tibia bone from adult ΔKlotho (*n* = 6) and control mice (*n* = 8). All data are presented as means ± SEM. *p < 0.05, **p < 0.01, *** *p* < 0.001. (PDF 39024 kb)

Additional file 2: **Figure S2.** Fiber types are not changed in ΔKlotho mice. (A) Representative immunofluorescence stainings of cross-sections from EDL of p56 ΔKlotho and control mice stained for DAPI (DNA, blue), laminin (white), MHCIIa and MHCIIb, or MHCIIa and MHCIIx, respectively. Scale bar = 50 μm. (B) Percentage of MHC type IIa and IIb positive myofibers on EDL cross-sections from ΔKlotho and control mice at p56. (ΔKlotho *n* = 3 mice, control *n* = 7 mice). (C) Percentage of MHC type IIa and IIx positive myofibers on EDL cross-sections from ΔKlotho and control mice at p56. (ΔKlotho *n* = 3 mice, control *n* = 6 mice). All data are presented as means ± SEM. (PDF 8407 kb)

Additional file 3: **Figure S3.** The differentiation of myoblasts from ΔKlotho mice is not affected in vitro. (A) Quantification of the average number of nuclei per myotube counted on 6 random regions of interest per condition after 5 days of differentiation (ΔKlotho *n* = 3 mice, control *n* = 4 mice). (B) Percentage of myogenin-positive nuclei of all nuclei within myotubes after 5 days of differentiation (ΔKlotho *n* = 3 mice, control *n* = 4 mice). (C) Differentiation index (percentage of myotubes with more than three nuclei) after 5 days of differentiation (ΔKlotho *n* = 3 mice, control *n* = 4 mice). (D) Distribution of classes of myotubes after 5 days of differentiation (ΔKlotho *n* = 3 mice, control *n* = 4 mice). (E) Immunoblot analyses of supernatants from primary myoblasts and lysate from a kidney from wt animals using an antibody directed against klotho showing sKlotho in the supernatant and in whole kidney lysates (as expected). (F) Ponceau stained membrane showing similar loading of concentrated supernatants from primary myoblasts isolated from ΔKlotho and control mice. All data are presented as means ± SEM. (PDF 7378 kb)

Additional file 4: **Figure S4.** Muscle stem cell function is impaired in adult ΔKlotho mice. (A) The activation potential (number of clusters per myofiber after 72 h of culture divided by the number of muscle stem cells per myofiber directly after isolation) (ΔKlotho *n* = 5 mice, control *n* = 7 mice). (B) Percentage of Pax7+/MyoD- cells within a cluster on myofibers isolated from p42 old mice. (ΔKlotho *n* = 5 mice, control *n* = 7 mice). (C) Percentage of Pax7+/MyoD+ cells within a cluster on myofibers isolated from p42 old mice. (ΔKlotho *n* = 5 mice, control *n* = 7 mice). (D) Percentage of Pax7−/MyoD− cells within clusters on myofibers isolated from p42 old mice. (ΔKlotho *n* = 5 mice, control *n* = 7 mice). All data are presented as means ± SEM. *p < 0.05. (PDF 511 kb)

Additional file 5: **Figure S5.** Addition of recombinant sKlotho protein rejuvenates aged muscle stem cells. (A) Addition of sKlotho to primary myoblasts reduces canonical Wnt signaling induced by addition of recombinant Wnt3a as evidenced by measuring levels of phosho-beta-catenin and non-phospho-beta-catenin, densitometric analysis of Fig. 6b after normalization to GAPDH, values are shown as normalization to control. (B) Myofibers with their adjacent muscle stem cells from young (4 months) and old (22–24 months) mice were cultured for 72 h with medium, recombinant soluble klotho (KL) protein, recombinant Dkk1 or a combination of both. The number of clusters per myofiber was normalized to young control. (*n* = 4 mice (young), *n* = 3 (old)). (C) The activation potential is increased in old mice when sKlotho protein (KL) is added (*n* = 4). (D) Myofibers with their adjacent muscle stem cells from young (4 months) and old (22–24 months) mice were cultured for 72 h with medium, recombinant soluble klotho (KL) protein, recombinant Dkk1 or a combination of both. The number of Pax7+/MyoD− cells per myofiber was normalized to young control. (*n* = 4 mice (young), *n* = 3 (old)). All data are presented as means ± SEM. *p < 0.05. (PDF 616 kb)

## Abbreviations

devMHC: Developmental myosin heavy chain
Dkk1: Dickkopf-related protein 1
EDL: Extensor digitorum longum
Fbxo32: F-Box Protein 32
FGF23: Fibroblast growth factor 23
Hox: Homeobox
IGF1: Insulin-like growth factor 1
JAK: Janus kinase
mKlotho: Membrane Klotho
MuRF: Muscle RING-finger protein
MyoD: Myogenic differentiation 1
Pax7: Paired box protein 7
sKlotho: Soluble Klotho
SPF: Specific pathogen free
STAT: Signal Transducer and Activator of Transcription proteins
TA: Tibialis anterior
TGF-β: Transforming growth factor β
Wnt: Wingless-related integration site
