# Supplementary figures and images for: Klotho expression is a prerequisite for proper muscle stem cell function and regeneration of skeletal muscle
Source: Skelet Muscle. 2018 Jul 4;8:20. doi: 10.1186/s13395-018-0166-x (PMC6030782; doi:10.1186/s13395-018-0166-x)

# Figure S1

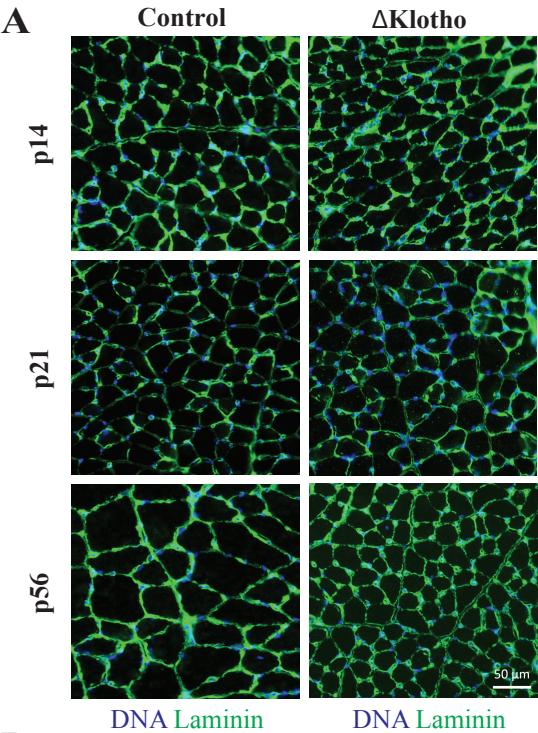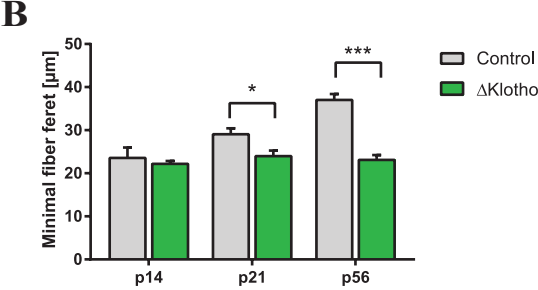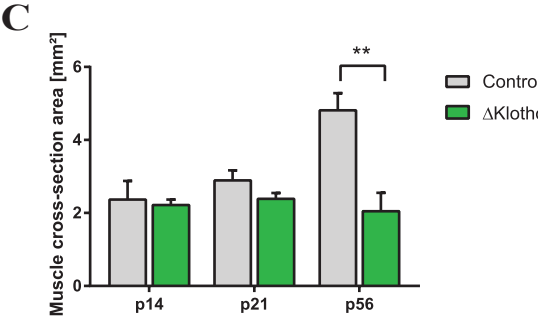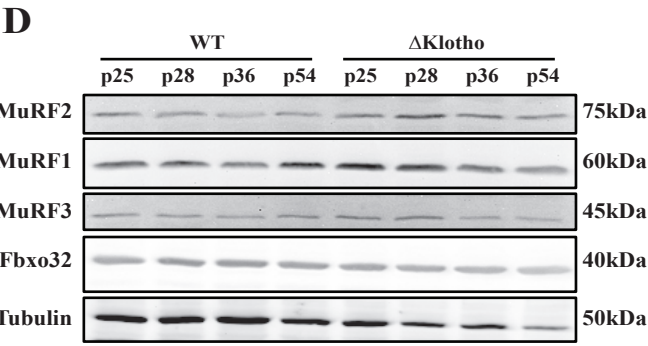

**E**

|     | MuRF1 | MuRF2 | MuRF3 | Fbxo32 |
|-----|-------|-------|-------|--------|
| p25 | 1,63  | 1,25  | 1,31  | 1,02   |
| p28 | 2,36  | 3,12  | 2,54  | 1,24   |
| p36 | 2,20  | 3,88  | 1,97  | 1,24   |
| p54 | 1,70  | 3,10  | 1,83  | 1,61   |

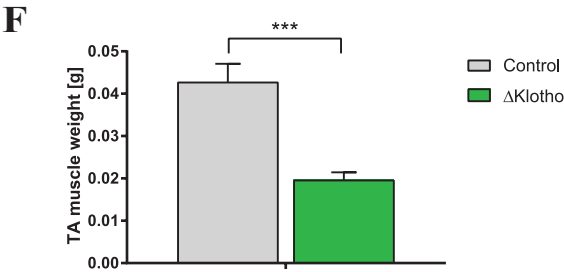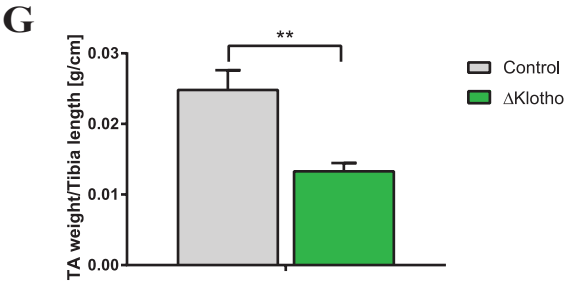

Supplement: Supplementary file 1 — Figure S1. ΔKlotho mice display signs of sarcopenia. (A) Immunofluorescence of cross-sections of TA muscles from ΔKlotho and control mice stained for DAPI (DNA, blue) and Laminin (green) at p14, p21, and p56. Scale bar = 50 μm. (B) Minimal fiber feret measured on the whole cross-sections from ΔKlotho and control TA muscles at p14, p21, and p56. (p14, p56 n ≥ 3 mice per genotype, p21 n = 3 mice per genotype). (C) Quantification of the cross-section area of the mid-belly region of TA muscles determined from sections from ΔKlotho and control at p14, p21, and p56. (p14, p56 n ≥ 3 mice per genotype, p21 n = 3 mice per genotype). (D) Immunoblot analyses of atrophy associated ubiquitin ligases in TA muscles from ΔKlotho and control littermates at different ages. (E) Fold expression of different ubiquitin ligases as shown in (D), values for ΔKlotho are normalized to control animal of the same age. (F) Muscle weight of tibialis anterior (TA) muscles from adult ΔKlotho (n = 6) and control mice, n = 8 (G) Muscle weight of tibialis anterior (TA) muscles normalized to the length of the tibia bone from adult ΔKlotho (n = 6) and control mice (n = 8). All data are presented as means ± SEM. *p < 0.05, **p < 0.01, *** p < 0.001. (PDF 39024 kb) [file 13395_2018_166_MOESM1_ESM.pdf]

Figure S2

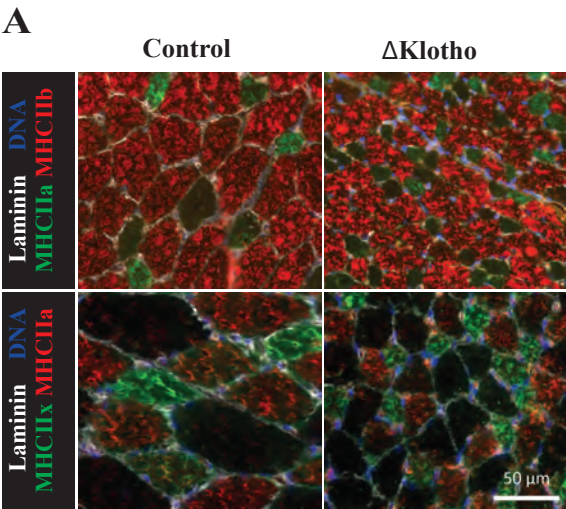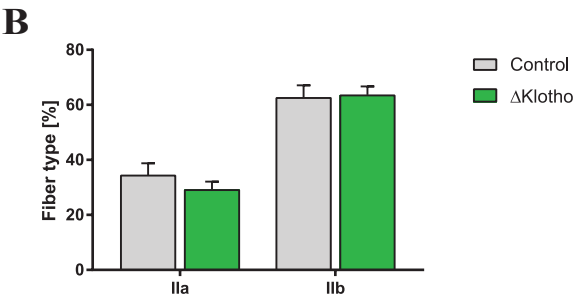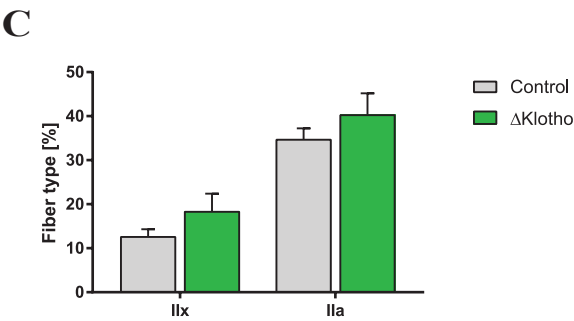

Supplement: Supplementary file 2 — Figure S2. Fiber types are not changed in ΔKlotho mice. (A) Representative immunofluorescence stainings of cross-sections from EDL of p56 ΔKlotho and control mice stained for DAPI (DNA, blue), laminin (white), MHCIIa and MHCIIb, or MHCIIa and MHCIIx, respectively. Scale bar = 50 μm. (B) Percentage of MHC type IIa and IIb positive myofibers on EDL cross-sections from ΔKlotho and control mice at p56. (ΔKlotho n = 3 mice, control n = 7 mice). (C) Percentage of MHC type IIa and IIx positive myofibers on EDL cross-sections from ΔKlotho and control mice at p56. (ΔKlotho n = 3 mice, control n = 6 mice). All data are presented as means ± SEM. (PDF 8407 kb) [file 13395_2018_166_MOESM2_ESM.pdf]

# Figure S3

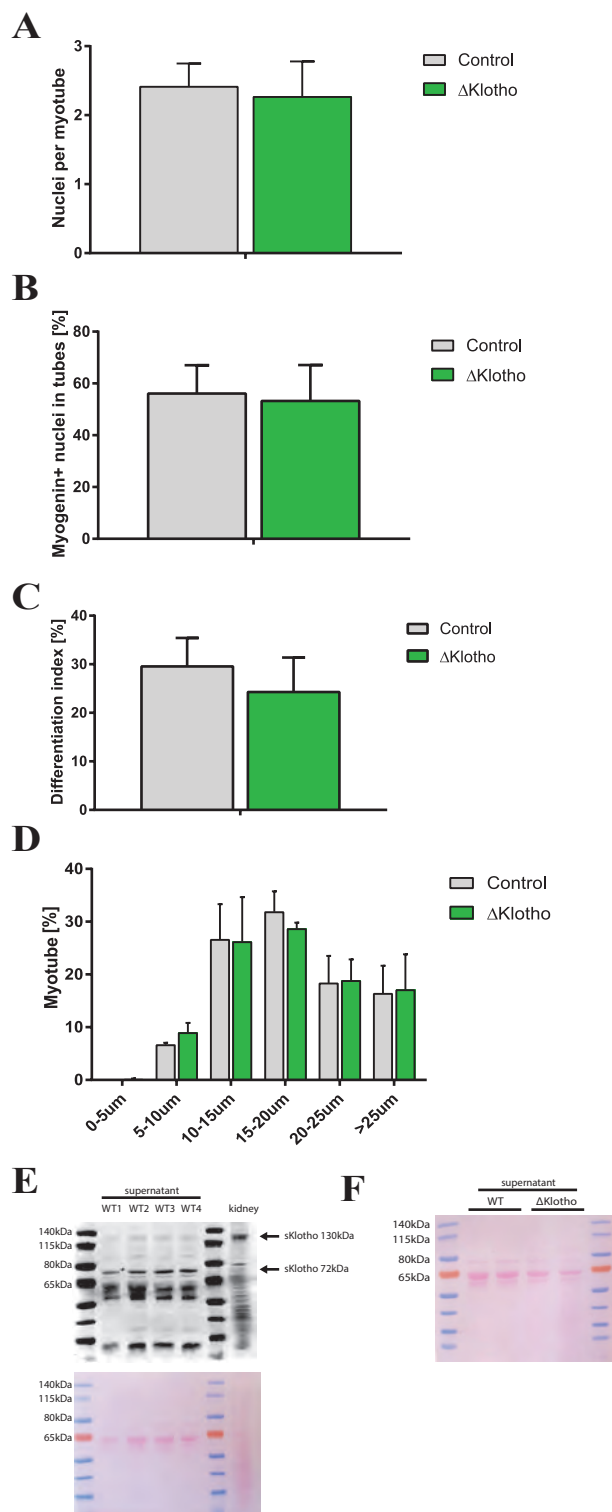

Supplement: Supplementary file 3 — Figure S3. The differentiation of myoblasts from ΔKlotho mice is not affected in vitro. (A) Quantification of the average number of nuclei per myotube counted on 6 random regions of interest per condition after 5 days of differentiation (ΔKlotho n = 3 mice, control n = 4 mice). (B) Percentage of myogenin-positive nuclei of all nuclei within myotubes after 5 days of differentiation (ΔKlotho n = 3 mice, control n = 4 mice). (C) Differentiation index (percentage of myotubes with more than three nuclei) after 5 days of differentiation (ΔKlotho n = 3 mice, control n = 4 mice). (D) Distribution of classes of myotubes after 5 days of differentiation (ΔKlotho n = 3 mice, control n = 4 mice). (E) Immunoblot analyses of supernatants from primary myoblasts and lysate from a kidney from wt animals using an antibody directed against klotho showing sKlotho in the supernatant and in whole kidney lysates (as expected). (F) Ponceau stained membrane showing similar loading of concentrated supernatants from primary myoblasts isolated from ΔKlotho and control mice. All data are presented as means ± SEM. (PDF 7378 kb) [file 13395_2018_166_MOESM3_ESM.pdf]

# Figure S4

**A**

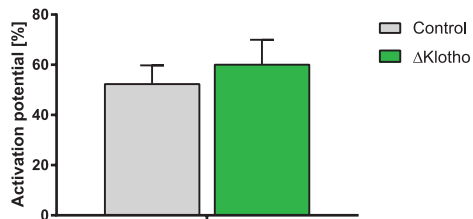

**B**

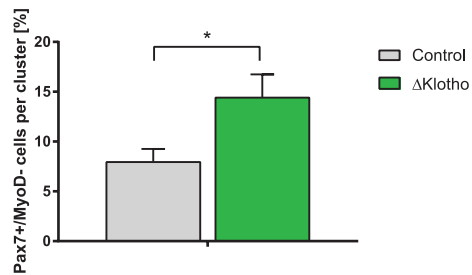

**C**

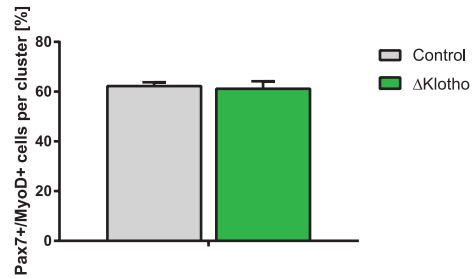

**D**

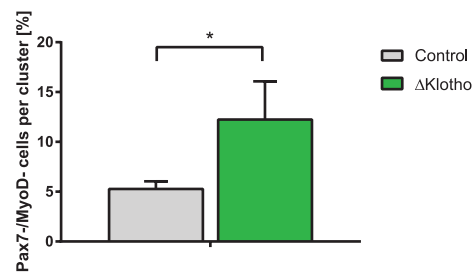

Supplement: Supplementary file 4 — Figure S4. Muscle stem cell function is impaired in adult ΔKlotho mice. (A) The activation potential (number of clusters per myofiber after 72 h of culture divided by the number of muscle stem cells per myofiber directly after isolation) (ΔKlotho n = 5 mice, control n = 7 mice). (B) Percentage of Pax7+/MyoD- cells within a cluster on myofibers isolated from p42 old mice. (ΔKlotho n = 5 mice, control n = 7 mice). (C) Percentage of Pax7+/MyoD+ cells within a cluster on myofibers isolated from p42 old mice. (ΔKlotho n = 5 mice, control n = 7 mice). (D) Percentage of Pax7−/MyoD− cells within clusters on myofibers isolated from p42 old mice. (ΔKlotho n = 5 mice, control n = 7 mice). All data are presented as means ± SEM. *p < 0.05. (PDF 511 kb) [file 13395_2018_166_MOESM4_ESM.pdf]

# Figure S5

**A**

|                | phospho-beta-Catenin | non-phospho-beta-Catenin |
|----------------|----------------------|--------------------------|
| control        | 1                    | 1                        |
| Klotho         | 1,29                 | 0,89                     |
| Wnt3A          | 0,58                 | 1,39                     |
| Klotho + Wnt3a | 0,79                 | 1,30                     |

**B**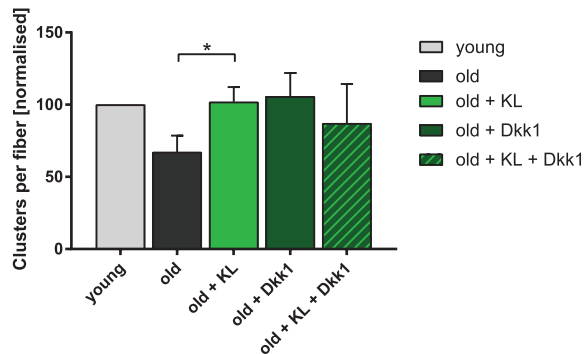**C**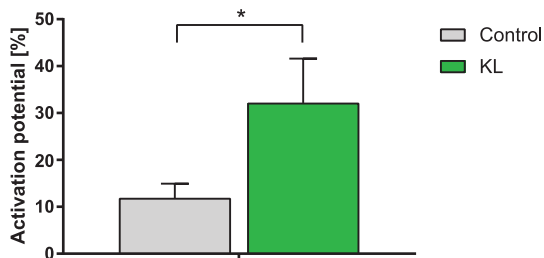**D**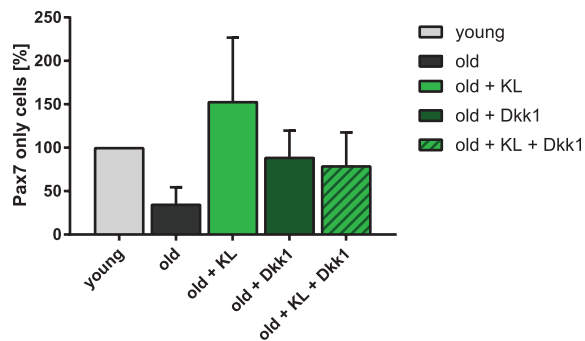

Supplement: Supplementary file 5 — Figure S5. Addition of recombinant sKlotho protein rejuvenates aged muscle stem cells. (A) Addition of sKlotho to primary myoblasts reduces canonical Wnt signaling induced by addition of recombinant Wnt3a as evidenced by measuring levels of phosho-beta-catenin and non-phospho-beta-catenin, densitometric analysis of Fig. 6b after normalization to GAPDH, values are shown as normalization to control. (B) Myofibers with their adjacent muscle stem cells from young (4 months) and old (22–24 months) mice were cultured for 72 h with medium, recombinant soluble klotho (KL) protein, recombinant Dkk1 or a combination of both. The number of clusters per myofiber was normalized to young control. (n = 4 mice (young), n = 3 (old)). (C) The activation potential is increased in old mice when sKlotho protein (KL) is added (n = 4). (D) Myofibers with their adjacent muscle stem cells from young (4 months) and old (22–24 months) mice were cultured for 72 h with medium, recombinant soluble klotho (KL) protein, recombinant Dkk1 or a combination of both. The number of Pax7+/MyoD− cells per myofiber was normalized to young control. (n = 4 mice (young), n = 3 (old)). All data are presented as means ± SEM. *p < 0.05. (PDF 616 kb) [file 13395_2018_166_MOESM5_ESM.pdf]
